# Dietary therapy in abdominal aortic aneurysm — Insights from clinical and experimental studies

**DOI:** 10.3389/fcvm.2022.949262

**Published:** 2022-09-21

**Authors:** Li Yin, Alexander Christopher Gregg, Alessandra Marie Riccio, Nicholas Hoyt, Zain Hussain Islam, Jungeun Ahn, Quang Le, Paranjay Patel, Mengxue Zhang, Xinran He, Matthew McKinney, Eric Kent, Bowen Wang

**Affiliations:** ^1^Department of Surgery, School of Medicine, University of Virginia, Charlottesville, VA, United States; ^2^School of Medicine and Health Sciences, George Washington University, Washington, DC, United States

**Keywords:** diet, abdominal aortic aneurysm, dietary therapy, dietary restriction (DR), nutrient-sensing pathway, gut microbiome

## Abstract

Abdominal aortic aneurysm (AAA) is a prevalent vascular disease with high mortality rates upon rupture. Despite its prevalence in elderly populations, there remain limited treatment options; invasive surgical repair, while risky, is the only therapeutic intervention with proven clinical benefits. Dietary factors have long been suggested to be closely associated with AAA risks, and dietary therapies recently emerged as promising avenues to achieve non-invasive management of a wide spectrum of diseases. However, the role of dietary therapies in AAA remains elusive. In this article, we will summarize the recent clinical and pre-clinical efforts in understanding the therapeutic and mechanistic implications of various dietary patterns and therapeutic approaches in AAA.

## Introduction

Abdominal aortic aneurysm (AAA) is a localized, progressive weakening and dilation of the aortic wall, primarily in the infrarenal segment. It is a common vascular disease in elderly populations (i.e., >65-year old), and up to 8% of males and 6% of females are estimated to develop AAA over a lifetime (1). While most cases are asymptomatic, AAA features a highly unpredictable disease course, which could culminate in the highly deadly rupture of the aneurysmal aorta (2). Ruptured AAAs have up to an 85% mortality rate, and thus far no single parameter or tool can robustly predict the risk of rupture (3). Owing to the expanded AAA screening in at-risk populations, an increasing number of diagnosed yet asymptomatic patients have been identified, albeit the majority of which are small-diameter AAAs. Unfortunately, these patients do not meet the criteria for open or endovascular repairs, which remain the standard of care (4). In these scenarios, a “watchful waiting” strategy is often employed to monitor the AAA growth rate overtime to triage patients that are considered stable; but due to the lack of effective pharmacotherapies to block AAA expansion, these patients are left unprotected from the risk of unpredictable and lethal rupture (5). The absence of therapeutic modalities alternative and complementary to surgical repairs has greatly hindered the success brought about by the AAA surveillance initiative, causing unnecessary anxiety to the patients as well as burdens to the healthcare system (6).

Strategies to counter AAA's modifiable risk factors have long been recommended as secondary preventative measures to reduce overall cardiovascular mortality risks (5). Aside from the non-modifiable risk factors such as advanced age, male gender, Caucasian race, and family history, modifiable risk factors offer potential avenues for risk reduction strategies (7–9). Tobacco smoking is arguable the strongest modifiable risk factor associated with AAA expansion and rupture, and smoking cessation has been widely and strongly recommended for all patients with AAA (10, 11). Other modifiable factors, including hypertension and atherosclerosis, have also been subjected to extensive studies. However, at present, none of the anti-hypertensive (e.g., beta blockers) or atherosclerosis medications (e.g., statins) have demonstrated clear therapeutic efficacies against AAA expansion and rupture (12).

The lack of definitive clinical benefits in the foregoing risk reduction strategies necessitates further efforts toward the first non-surgical treatment of AAA. Over the past two decades, numerous FDA-approved drugs have been repurposed for treating AAAs, yet none have yielded any clinical success (12). The most prominent example is doxycycline, a previously approved tetracycline antibiotic for anti-bacterial infections that was hypothesized to possess potent anti-AAA efficacy due to its additional benefits in inhibiting proteolytic enzyme activities and inflammasome activation. Unfortunately, after almost twenty years of active investigations, the two randomized clinical trials — the N-TA3CT trial in the US and the PHAST trial in Netherland — led to the conclusion that doxycycline had no benefits against, if not further exacerbating the expansion of small-diameter AAAs (13, 14). Other promising candidate drugs, such as metformin and sirolimus, are still far from the clinical utility at the current stage (15, 16). As such, other alternative strategies are urgently needed to address the unmet clinical need, that is a safe and effective non-invasive management of AAA.

**Figure 1:**
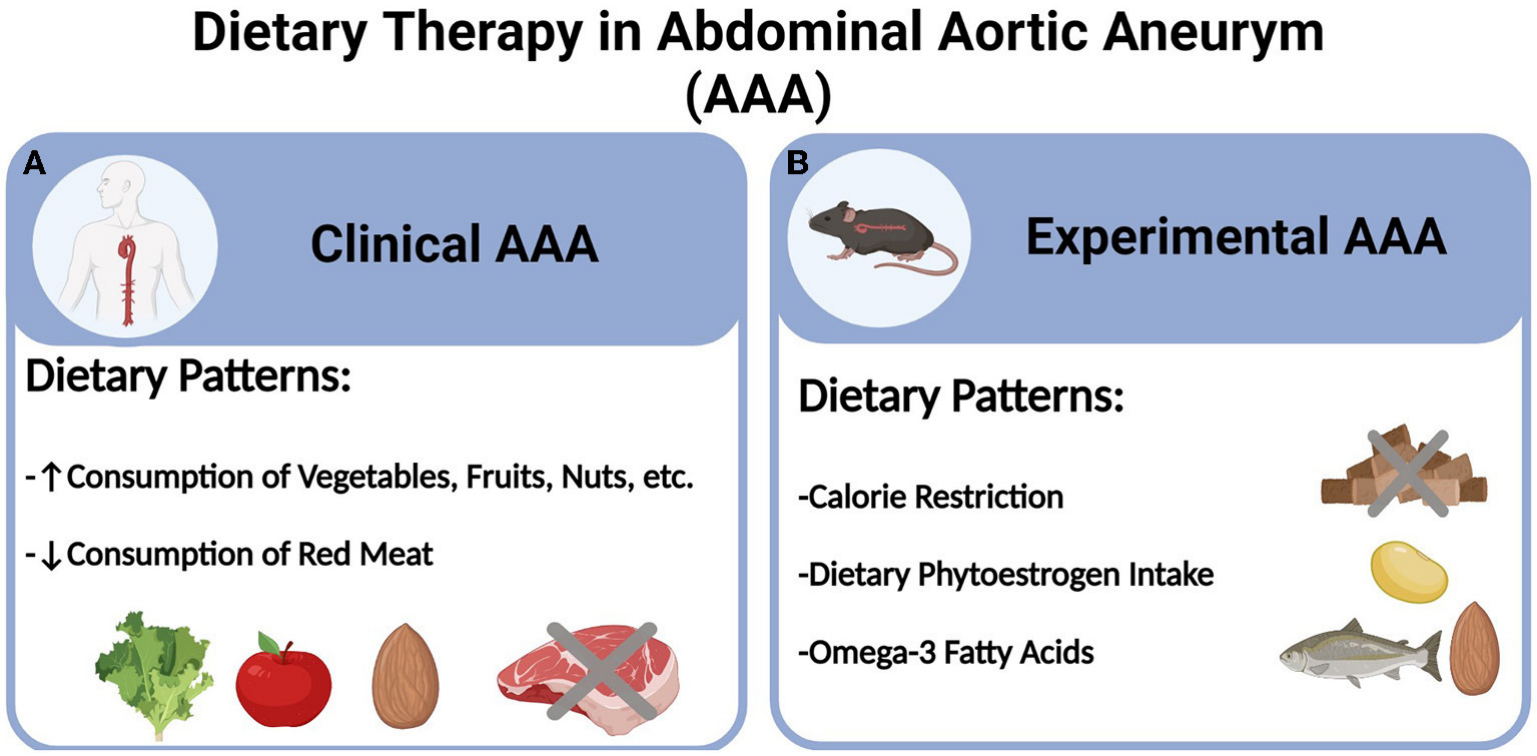
Dietary therapy in abdominal aortic aneurysm (AAA). **(A)** Clinical AAA and **(B)** Experimental AAA.

In light of the aforementioned challenges and obstacles, recent years have witnessed a surging interest in pursuing lifestyle changes for AAA management. While smoking cessation and physical exercise both have shown promising benefits in reducing AAA risks, there is a scarcity of studies concerning the role of healthy dietary patterns, at both clinical and preclinical levels (10, 17, 18). Especially with the ever-increasing benefits of various dietary regimens against cardiovascular diseases as recently reported (19, 20), understanding the therapeutic and mechanistic implications of certain dietary patterns would hold significant value in informing the future guideline of AAA management. Herein, we will summarize the recent progress in dietary therapies for AAA based on epidemiological and experimental evidence, (see Figure 1). Additionally, we will discuss the perspectives of emerging dietary regimens and potential molecular basis.

## Clinical evidence supporting dietary regimens in AAA

In contrast to surgical repairs and drug treatments, dietary therapies are uniquely advantageous in multiple aspects, ranging from accessibility to the lack of invasiveness. For the management of cardiovascular diseases and metabolic syndromes, healthy dietary patterns have long been recommended to patients with proven benefits (19–22). However, in the case of AAA, the number of clinical studies examining the role of dietary patterns is sparse, with the epidemiological data almost exclusively derived from the following cohort studies: the Life Line Screening study, Atherosclerosis Risk in Communities (ARIC) study, the Malmö Diet and Cancer Study (MDCS), the Cohort of Swedish Men and the Swedish Mammography Cohort, and the Health In Men Study (HIMS), and most recently, the Brazilian Cardioprotective Nutritional Program Trial (23).

The first epidemiological evidence concerning dietary elements came from the Life Line Screening study in the US. In this retrospective cohort of 3.1 million patients, Kent et al. reported that consumption of nuts, vegetables, and fruits at a frequency of >three times per week were associated with reduced risk of AAA, as was the case for exercise (≥1 time per week) and smoking cessation (7). However, in this questionnaire study, fruits and vegetables were combined, thus their respective link to AAA risk was not discerned.

Built upon the seminal work from Kent et al., Stackelberg et al. continued to investigate dietary factors, particularly fruit and vegetable consumption, using the prospective Cohort of Swedish Men and Mammography Cohort (24). Surprisingly, while consumption of fruit was found to be negatively associated with the risk of AAA (particularly the ruptured ones), no link was established for vegetable consumption. In a follow-up study, the same team reported their finding that moderate alcohol consumption was inversely associated with AAA (25).

The role of dietary fiber and vegetable intake in AAA was not established until the reports from Harring et al. in 2018 and Bergwall et al. in 2019. In the former study, the ARIC study cohort was investigated regarding the association between AAA risk and their adherence to the Dietary Approaches To Stop Hypertension (DASH) dietary patterns (26). In addition to fruits, nuts, and legumes, high consumption of vegetables, whole grains, and low-fat dairy were revealed to be associated with decreased AAA burden, respectively. The latter study, conducted in the MDCS cohort, showed that linear protective associations between high intakes of fruits, berries, vegetables with AAA. Surprisingly, potato consumption was positively associated with AAA risk (27). Of particular note in both studies is the lack of statistically significant correlation between AAA risk and salt consumption, contradicting a prior population screening study in 11,742 elderly Australian males (28). Considering the uncertain clinical benefits of anti-hypertensive medications and the increasing popularity of the DASH dietary style, further experimental and clinical investigations are warranted to clarify the interventional value of such dietary patterns with restricted salt intake.

Similar to the DASH diet studied in the ARIC cohort, other similar dietary patterns have also been studied, including the anti-inflammatory diet and Mediterranean diet (29, 30). Similarly, diets high in antioxidant contents have also been recently reported to be associated with reduced AAA risks (23, 31). All these dietary patterns feature similar compositions, such as high consumption of fruit, vegetables, nuts, legumes, wholegrains, fish, and low consumption of red and processed meat. From a utility perspective, studying dietary patterns and eating styles may hold more translational value than focusing on individual food item consumption, as the former can encompass a variety of healthy dietary elements as aforementioned.

## Preclinical studies concerning dietary patterns in AAA

### Dietary therapies in experimental models

Notwithstanding the growing interest and epidemiological evidence, experimental studies concerning dietary therapies are very limited. Based on our literature search, three types of dietary regimens have been identified with preclinical therapeutic efficacies in murine models of AAA, i.e., calorie restriction, dietary phytoestrogen intake, and consumption of long-chain Omega-3 polyunsaturated fatty acids (PUFAs).

#### Calorie restriction

Calorie restriction is a dietary regimen that consists of decreased calorie intake without causing malnutrition and is often prescribed to achieve weight loss. Experimental data derived from rodent and non-human primate models support a pleiotropic role of calorie-restricted diets in mediating a myriad of health benefits, most notably in prolonging lifespan (32, 33). Data from the calorie restriction and cardiometabolic risk (CALERIE) study revealed a clear benefit of calorie restriction in reducing cardiometabolic risk factors (34). To date, only two experimental reports concerning the impact of such dietary patterns are available. Liu et al. observed profound mitigation of AAA formation using a calorie-restricted diet in angiotensin II (AngII)-infused, Apoe-/- mice (35). Additionally, they further found that sirtuin 1 (SIRT1), but not other metabolic/energy sensors such as SIRT3, mechanistic target of rapamycin (mTOR), and AMP-activated protein kinase α (AMPKα), was the main mediator of the aortic benefits exerted by calorie restriction. Such AAA-protective effect was later recapitulated using the same murine model and dietary regimen, and an alternative mediator, p53, was discovered to contribute to the aforementioned phenotype, possibly through maintaining mitochondrial bioenergetics (36).

#### Phytoestrogen diet

Phytoestrogens are a group of plant-derived chemicals with structural and functional similarities to 17-β-estradiol, an estrogen with a presumed protective role against AAA. The primary sources of phytoestrogen intake are through consumption of soy and many other legumes, which have been previously linked to reduced AAA risk. Using a murine model with topical elastase application, Lu et al. showed that a phytoestrogen-rich diet could effectively ameliorate AAA development in male but not in female mice (37). In line with this observation, supplementation with the phytoestrogen Daidzein attenuated AngII-induced AAA in murine models (38). However, a follow-up study by Fashandi et al. using a modified AngII model failed to demonstrate any impact of a high-phytoestrogen Western diet on AAA rupture rates or survival (39). The inconsistency amongst these studies could be potentially attributed to the different experimental models, dietary regimens, and the phytoestrogen level/compositions in different chows, as demonstrated in the work from Lu et al. Considering the striking gender dimorphism in AAA, future experimental efforts are poised to address the question concerning the link among dietary phytoestrogen, endogenous estrogen, and AAA risk (40).

#### Omega-3 PUFA-rich diet

Increased consumption of the PUFAs (e.g., Omega-3) — often deemed as the healthy fats — is a key component of the aforementioned diets (e.g., Mediterranean diet) featuring high consumption of fish and nuts. Indeed, numerous Omega-3 PUFAs have been studied in murine models, in which dietary supplementation could reduce AAA development (41–43). While small-scale clinical studies suggest a potential correlation between Omega-3 PUFAs and reduced AAA risk as well as early benefits in improving pre-AAA pathologies (i.e., aortic stiff), further studies are warranted to fully elucidate the role of such dietary component(s) in the clinical management of AAA (44, 45).

### AAA-promoting diets in experimental models

In stark contrast to the paucity of experimental studies concerning dietary therapies, a plethora of literature are available, detailing the pathophysiological/phenotypic impacts upon experimental AAA pathogenesis. Most notable examples include dietary patterns that recapitulate AAA's established risk factors, such as high-fat diets (atherosclerosis/hypercholesterolemia) (46, 47), high-salt consumption (hypertension), excessive supplementation with homocysteine, or methionine (red meat consumption and hyperhomocysteinemia), etc (48–50).

## Perspectives

### Emerging dietary regimens to be explored in preclinical AAA models

#### Protein restriction

Similar to calorie restriction, protein restriction has also been shown to help prolong lifespan in fruit flies and mice (51, 52). In epidemiological studies, reduced intake of proteins, especially red meat consumption, has been related to reduced risk of all-cause mortality in populations aged 50–65 years. However, in elderly populations aged above 65 years that are also at risk of AAA, low protein intake rather increased, whereas high protein consumption reduced all-cause mortality (53). These observations highlight the duality behind dietary restrictions on general nutrient intake: on the one hand, such strategies have yielded pleiotropic benefits; but on the flip side, concerns of malnutrition and frailty have been persistently plaguing their widespread adoption and long-term adherence, particularly for the AAA-prone aged populations that are more susceptible to side effects like sarcopenia. Interestingly, recent studies suggest that short-term dietary restrictions, such as pre-operative protein restriction under inpatient settings, may present an alternative, viable path as dietary preconditioning to safely capitalize on the benefits of such dietary therapies. Indeed, mounting preclinical evidence has pointed to the role of pre-operative protein restriction in reducing surgical stress as well as vascular (re)stenosis. Inspired by the safety data from an exploratory trial in elective carotid endarterectomy patients (54), another trial is currently underway to chart the baseline information from healthy subjects, with the ultimate goal of comparing with patients undergoing AAA surgical repair in a future study (NCT03995979). However, sufficient protein intake has also been suggested to be critical in post-operative recovery, and hence cautions should be noted concerning the frailties caused by protein restriction (55, 56).

#### Amino acid restriction

Unlike calorie or protein restriction, limiting the intake of amino acid(s) has been shown to produce similar health benefits in preclinical studies without significant risks of malnutrition. Clinical studies further established the safety profile as well as the early efficacy of a methionine-restricted diet in healthy and cancer patients, with additional randomized clinical trials currently ongoing (57, 58). While restrictions of single (e.g., methionine) or multiple (e.g., branch-chained amino acids) amino acids have been studied in experimental models and even human subjects of other cardiometabolic diseases, their implications in AAA have not been reported (59–63). Considering the clinical association between certain amino acids/metabolites (e.g., homocysteine, methionine) and AAA risk, it is of significant translational value to pursue the preclinical impacts of such dietary restriction patterns in future studies.

#### Intermittent fasting

Intermittent fasting is a dietary pattern that features time-restricted eating with or without reducing calorie intake. Due to its reduced risk of malnutrition and ease of adherence, intermittent fasting has garnered significant popularity during the past decade as an alternative regimen. Clinical studies have suggested beneficial associations of intermittent fasting with cardiometabolic risks, albeit long-term outcomes are still pending (64, 65). In light of the aortic protection exerted by calorie restriction in murine AAA models, it is reasonable to postulate whether a similar effect could be observed with intermittent fasting. However, it is worth noting that current intermittent fasting protocols are highly variable, thus making comparisons amongst different studies difficult. A recent murine study further demonstrated that the circadian schedules of the time-restricted eating could critically determine the outcomes of different fasting protocols, which informs the challenges and critical parameters to be considered when designing future preclinical studies in AAA (66).

### Emerging diet-mediated mechanisms potentially implicated in AAA

There is no pathway or molecular basis that could completely account for the health benefits of a given diet. Past studies have unveiled various mechanisms implicated in the aforementioned dietary regimens, such as overall energy expenditure reduction, antioxidant supplementation, increased production of hepatokines (e.g., FGF21) (67–71), etc. Due to the scarcity of relevant literature on AAA, we will focus on two emerging dietary mechanisms that are recently implicated in AAA pathophysiologies while more in-depth investigations are still needed.

#### Metabolism/nutrient sensors

Studies from other disciplines have uncovered a series of sensor proteins and pathways for cellular metabolism, energy expenditure, and certain nutrient cues. Amongst the sensors known thus far, the NAD+-dependent protein deacetylase sirtuin family member SIRT1 is the most notable example. Not only has SIRT1 been established to negatively modulate AAA pathogenesis (35, 72), experimental evidence further revealed its critical role in mediating the benefits of calorie restriction in mitigating AAA risk. Similarly, deletion of SIRT3, another sirtuin member, exacerbated, whereas its overexpression mitigated aneurysmal formation and progression (73). Another kinase commonly associated with dietary restriction is AMPKα, the activation of which has been the presumed mode of action behind established therapeutic regimens such as calorie restriction and metformin. Indeed, although no definitive link has been made between AMPKα and any diet-mediated AAA mitigation, data in murine models did suggest a protective role of AMPKα against AAA formation (74, 75); and while metformin, a calorie restriction mimetic therapy recapitulated the therapeutic benefits, AMPK blockade effectively abolished the protection against AAA (15, 74, 76).

mTOR and general control non-derepressible 2 (GCN2) constitute the only two identified sensors for amino acids (77). While mTOR is activated by recognizing the presence and intracellular level of amino acids, GCN2, on the other hand, senses amino acid starvation (78, 79). Both mTOR (inactivation) and GCN2 (activation) have been implicated in the cardiometabolic benefits of dietary restrictions (60, 80, 81). While chronic activation of mTOR was recently reported to drive aortic degeneration and hence aneurysmal formation, the specific role of GCN2 remains unknown in AAA (82). Further studies concerning GCN2 and amino acid restriction are poised to determine the clinical utility of such innovative dietary therapies.

#### Gut microbiome

You are what you eat. This is particularly true for the gut microbiome, as all the aforementioned diets have been shown to modulate gut microbiota (83–88). Although largely neglected as “bystanders” for a long time, the gut microbiome has been increasingly recognized to contribute to a wide range of biological and pathological processes (89). Only very recently were alterations of gut microbiota reported in experimental and clinical subjects with AAA, suggesting a microbial dysbiosis that is yet to be elucidated in aneurysm (90, 91). Although no studies thus far have investigated the exact role of diet-induced microbiota in AAA, it is highly plausible that certain dietary regimens could impact AAA pathogenesis *via* cultivating distinct microbiome compositions and hence driving a shift toward beneficial microbiota-derived metabolic compositions. In fact, some microbiota-derived metabolites that are profoundly inhibited upon dietary restrictions (57, 92, 93), such as trimethylamine N-oxide (TMAO), were recently revealed to contribute to AAA development (94).

## Closing remarks

The past decade has witnessed tremendous progress in dietary therapies for the management of cardiometabolic diseases and cancer, yet their therapeutic and mechanistic implications in AAA are still elusive. The current study provides the first comprehensive review of preclinical and clinical evidence supporting the potential adoption of certain dietary patterns in preventing and managing AAA. Also discussed are selected emerging dietary regimens as well as diet-mediated mechanisms, in which further investigations are still pending. We envision that future research efforts will be increasingly dedicated to a better understanding of as well as translational development of the first non-surgical management of AAA in the form of dietary therapies.

## Author contributions

LY and BW conceived the manuscript. AG, AR, NH, ZI, JA, QL, PP, MZ, XH, MM, and EK conducted the literature search and contributed to the drafting and editing of the manuscript. ZI, PP, and MM created the graphical abstract using BioRender.com. All authors contributed to the article and approved the submitted version.

## Funding

This work was supported by the National Institute of Health (NIH) grant R01HL162895 (to BW).

## Conflict of interest

Author BW is the corresponding author, the coordinator for this research topic, and has recused from the peer-review and editorial processes. The remaining authors declare that the research was conducted in the absence of any commercial or financial relationships that could be construed as a potential conflict of interest.

## Publisher's note

All claims expressed in this article are solely those of the authors and do not necessarily represent those of their affiliated organizations, or those of the publisher, the editors and the reviewers. Any product that may be evaluated in this article, or claim that may be made by its manufacturer, is not guaranteed or endorsed by the publisher.
